# Effects of Nicotine Gum Administration on Vision (ENIGMA-Vis): Study Protocol of a Double-Blind, Randomized, and Controlled Clinical Trial

**DOI:** 10.3389/fnhum.2020.00314

**Published:** 2020-09-08

**Authors:** Thiago P. Fernandes, Jeffery K. Hovis, Natalia Almeida, Jandirlly J. S. Souto, Thiago Augusto Bonifacio, Stephanye Rodrigues, Gabriella Medeiros Silva, Michael Oliveira Andrade, Jessica Bruna Silva, Giulliana H. Gomes, Milena Edite Oliveira, Eveline Holanda Lima, Maria Eduarda Gomes, Marcos V. A. Junior, Mariana Lopes Martins, Natanael A. Santos

**Affiliations:** ^1^Department of Psychology, Federal University of Paraiba, João Pessoa, Brazil; ^2^Perception, Neuroscience and Behaviour Laboratory, Department of Psychology, Federal University of Paraiba, João Pessoa, Brazil; ^3^School of Optometry and Vision Science, University of Waterloo, Waterloo, ON, Canada; ^4^Department of Psychology, State University of Minas Gerais, Belo Horizonte, Brazil; ^5^Medical Sciences College, João Pessoa, Brazil; ^6^Department of Speech Therapy, Federal University of Paraiba, João Pessoa, Brazil

**Keywords:** clinical trial, visual processing, nicotine gum, smoking, nicotine, psychopharmacology

## Abstract

Studies reported that tobacco addiction was related to visual impairments, but one unresolved issue is whether the impairments are related to the many compounds existing in the cigarettes or to the effects of nicotine. On the other hand, nicotine gum can be used as replacement therapy or as a neuroprotective agent for some diseases. The main purpose of this controlled trial is to investigate the effects of nicotine gum on vision. The ENIGMA-Vis trial aims to compare two dosages of nicotine gum (2 and 4 mg) and a placebo gum in a randomized, double-blind, placebo-controlled trial of 100 participants to be allocated into a single group assignment of repeated measures (two studies; *N* = 50 for each one). Eligibility criteria are healthy non-smokers not diagnosed with substance abuse and without an acute or chronic medical condition. Intervention will last three sessions for each participant with a window frame of 1 week per session. Study outcomes are (1) short-term effects of nicotine gum on contrast sensitivity; (2) short-term effects of nicotine gum on chromatic contrast discrimination; and (3) whether demographics, body mass index, or serum cotinine predicts response of visual processing. This study addresses an important gap in the effects of nicotine on vision. One of the main takeaways of this study is to understand the effects of nicotine on contrast sensitivity and chromatic contrast discrimination. This information will provide a further understanding of how nicotine interacts with early visual processes and help determine how the different components present during smoking can affect vision.

**Clinical Trial Registration Number:** RBR-46tjy3.

## Introduction

Smoking is one of the world’s major public health problems. Chronic tobacco consumption, most of the time inhaled by cigarettes, is recognized as a substance use disorder (World Health Organization [WHO], 2015), and nicotine in cigarettes has long been associated with rapid and intense physical dependence (Jasinska et al., 2014). Chronic tobacco smoking is associated with a variety of health problems, including a higher incidence of coronary heart disease, cerebrovascular disease (e.g., stroke and transient ischemic attacks), and vascular diseases (e.g., aortic aneurysm and atherosclerosis) (Fernandes et al., 2018a). Furthermore, oxidative stress caused by chronic smoking is a risk factor for macular degeneration (i.e., a visual loss) (Velilla et al., 2013). Although cigarettes contain other harmful compounds, nicotine is the primary psychoactive compound in them and the most related to tobacco dependence.

A review of studies investigating the effects of smoking on vision provides a reference for examining the effects of nicotine on vision. Studies have shown that more prolonged duration of smoking, in years, resulted in an increased risk of visual loss (e.g., tobacco amblyopia and age-related macular degeneration) as observed using some techniques (e.g., event-related potentials and contrast sensitivity) (Rizzo and Lessell, 1993; Pickworth et al., 1997; Erskine et al., 2004; Fernandes et al., 2018b). The current consensus is that smoking affects vision (Conrin, 1980; Kunchulia et al., 2014; Arda et al., 2015; Fernandes et al., 2018b,c; Silva et al., 2020). However, this topic deserves further investigation as there are questions about the extent of how cigarette affects the (i) lens of the eyes, (ii) pupil, (iii) retina, and (iv) visual pathways. Also, many questions regarding the effects of daily cigarette consumption, duration, and intensity of smoking, age, sex, and the type of cigarette on vision remain to be addressed. More research is required to understand if these impairments are related to the compounds of cigarettes or nicotine intake. The roles of both tobacco smoking and nicotine addiction (due to smoking use) and the interactions with the nicotinic acetylcholine receptors (nAChRs) remain a matter of ongoing discussion.

Nicotine binds to the nAChRs and changes the receptor’s conformation, opening the ion channels and allowing sodium and calcium to enter causing depolarization and facilitating the release of a variety of neurotransmitters throughout the brain via α7 and β2 subunits (Dani, 2015). Low concentrations of nicotine (equivalent to smoking one or two cigarettes; Russell et al., 1976) facilitate the release of glutamate, gamma-aminobutyric acid (GABA), and dopamine neurotransmitters. The release of these neurotransmitters, in low concentrations, regulates synaptic plasticity in several areas of the central nervous system and has been implicated with improvement in some cognitive functions like memory and attention (Ellis et al., 2006; Hasselmo, 2006). Nicotine exposure seems to produce short-term effects on the release of some neurotransmitters and improve brain functions. These positive short-term effects have led to using nicotine as a neuroprotective agent for some diseases (e.g., Parkinson’s disease) (Quik et al., 2012); on the other hand, chronic tolerance (e.g., via chronic smoking) leads to the development of a long-lasting negative feedback loop on the release of neurotransmitters, decreasing the number of nAChRs due to desensitization (Halappanavar et al., 2013).

Nicotinic receptors are found throughout the visual system including the retina, lateral geniculate nucleus (LGN; both ventral and dorsal regions), the suprachiasmatic nucleus, primary visual cortex, fusiform gyrus, and prefrontal cortex (Paterson and Nordberg, 2000; Levin, 2001; Metherate, 2004; Govind et al., 2009). The role of the nAChRs within the retinal circuitry is still being worked out. However, the homomeric and heteromeric nAChRs (mostly α2–α7 and β2 subunits) have been found on the cones and rods photoreceptors (e.g., cell body, synaptic body, and synaptic terminals). nAChRs are also expressed in horizontal cells (e.g., dendrites and axons), bipolar cells (e.g., dendrites), amacrine cells (e.g., dendrites), and the ganglion cells (e.g., dendrites, cell bodies, and axons). Most of the nAChRs subtypes (from α2 to α8 and from β2 to β4) are found in these cells, but there is a greater heterogeneity in their expression. In the intrinsically photoreceptive retinal ganglion cells (ipRGCs), the α/β combination is expressed on their dendrites; however, the role of the nAChRs in the ipRGCs is still unclear (Paterson and Nordberg, 2000; Neal et al., 2001; Iwamoto et al., 2013; Dani, 2015).

The retinal circuitry carries out early processing of luminous contrast (achromatic) and chromatic information. The information from the retina is relayed to the LGN via three different pathways on the basis of where they project into the LGN. The parvocellular pathway is believed to carry both luminous contrast information for fine detail along with chromatic information based on the long- (L) and middle- (M) wavelength sensitive cones. The magnocellular pathway relays luminous contrast information for low-contrast, coarse (or large) detail in their cells (Govind et al., 2009; Fernandes et al., 2018c). The koniocellular pathway carries chromatic information on the basis of the short (S) wavelength sensitive cone and both the M- and L-cone responses. The pathways are maintained from the LGN to the visual cortex, where the color and luminous contrast information is combined to provide the visual perception (Cornsweet, 2012). The majority of the nAChRs in the LGN are expressed in the parvocellular pathway, but nicotine also has an affinity for the magnocellular and koniocellular pathways (Govind et al., 2009). Acetylcholine and nicotinic inputs modulate some features of stimulus like orientation and direction (Sillito and Kemp, 1983; Roberts et al., 2005; Zinke et al., 2006).

Because nAChRs are present throughout the visual pathway, both basic chromatic and luminous contrast perception could be affected. Nevertheless, it is unclear as to whether the extent of these processes would be differently affected. The result that the majority of nAChRs are located within the parvocellular pathway suggests that color information along the red–green (L-cone—M-cone) dimension could be affected more than along the blue–yellow dimension (S-cone pathway), and contrast sensitivity for high spatial frequencies could be affected more than contrast sensitivity at low spatial frequencies. This hypothesis assumes that the net result of nicotine at the retinal level does not differentially affect the inputs into the three LGN pathways. However, it is unclear whether greater heterogeneity in the expression of retinal nAChRs subunits would play a role in these inputs. Our study attempts to investigate, without establishing a causal relationship, whether the achromatic and chromatic contrast discrimination will be differentially affected, for example.

The primary use of nicotine is for nicotine replacement therapy in order to reduce withdrawal symptoms that occur during smoking cessation. Methods of administration include gum, lozenges, transdermal patches, nasal spray, and inhaler. The gum yields blood nicotine levels, similar to those after smoking cigarettes (Russell et al., 1976). The choice of using nicotine gum instead of other methods of nicotine administration was related to its low cost, fast absorption (compared to transdermal patches), high bioavailability, easy consumption, availability in low doses (2-mg and 4-mg), and fewer side effects (Fagerström et al., 1993; Aslani and Rafiei, 2012).

The effects of nicotine gum on vision are currently unclear. To this date, only two studies investigated the effects of nicotine gum on the visual system. Varghese et al. (2011) investigated the effects of nicotine gum in 10 healthy non-smokers. Their main results indicated that a 2-mg dose decreased amplitude in dark-adapted b-wave but had no effect in light-adapted b-wave. Similarly, a 4-mg dose decreased dark-adapted b-wave but increased light-adapted b-wave amplitudes. The b-wave reflects changes in primarily the bipolar cells activity and also the Müller glial cells, amacrine cells, and ganglion cells (see Silverstein et al., 2020, for a review). The increase in the amplitude could be due to nicotine increasing the release of dopamine in the retina. Increasing retinal dopamine does (or could) increase the amplitudes of the b-wave (S. Silverstein, e-communication), which could explain why the cone-mediated photopic b-wave increased; however, it would not explain the reduction in the rod-mediated scotopic b-wave unless dopamine was an inhibitory neurotransmitter within the rod pathways. Nevertheless, the perceptual consequences of these changes are uncertain.

The other study was investigating how nicotine affected color perception. Naser et al. (2011) observed that the use of nicotine gum reduced the total error score (TES) of the Farnsworth–Munsell Hue (i.e., improved chromatic discrimination) and decreased the threshold for detection of a red spot on white background in two healthy non-smokers. The improvement in the TES was larger for the 4-mg dosage compared with the 2-mg dose. On the other hand, the change in the red light detection threshold was larger for a 2-mg dose than a 4-mg dose.

One needs to be careful when interpreting the outcomes of these two experiments because the nicotine may not have been at the peak concentration within the system. Varghese et al. (2011) tested individuals after 30 min of nicotine exposure. As reported by Russell et al. (1976), the peak occurs between 15 and 40 min, after the beginning of the chewing. Thus, conducting an experiment after 30 min of nicotine gum administration could be outside the peak concentration. Naser et al. (2011) did not report the time window. Other limitations include their sample sizes were small, and neither experiment used a blind, controlled design.

The two studies on nicotine and vision suggest that nicotine could enhance photopic vision; however, studies on smoking suggest that nicotine, or another component in tobacco smoke, could impair photopic visual processes slightly by decreasing the pupil size and thereby reducing retinal illumination (Kunchulia et al., 2014; Erdem et al., 2015).

The Effects of Nicotine Gum on Vision Administration (ENIGMA-Vis) is, to the best of our knowledge, the first randomized controlled clinical trial that aims to develop a framework to investigate the effects of nicotine gum on basic visual processing. Here, we discuss the rationale, methods, and design of our ongoing trial. The main aim is to test if individuals have improvements in early-stage visual processing from nicotine gum administration (Levin, 2001). The primary hypothesis guiding our trial is that individuals using higher doses of nicotine gum will show better visual processing. The secondary hypotheses are that (a) differences in visual performance are mediated by the body mass index (BMI), sex (e.g., metabolism for nicotine is different between males and females) but not for other demographic data (e.g., age); and (b) differences in visual performance are mediated by the serum cotinine concentration.

This protocol is divided into two studies: the first study investigates contrast sensitivity, using the Metropsis software in healthy non-smokers. The gratings will be oriented vertically at spatial frequencies ranging from 0.2 to 20 cycles per degree so that the resolution capabilities of both the parvocellular and magnocellular pathways are assessed. The second study investigates chromatic contrast discrimination in healthy non-smokers using the Cambridge Colour Test (CCT). This test will measure discrimination along the individual cone dimensions and overall color discrimination relative to three different reference backgrounds. The measurements along the cone dimensions would help determine whether the three chromatic signals are differentially affected, and the overall discrimination would be a replication and/or an extension of the Naser et al. (2011) experiment. Findings obtained from this study will help to provide motivation for follow-up studies (e.g., comparing acute with chronic use of nicotine). The findings may help refine public health policies regarding tobacco use disorder, given the significance for public health. For example, if healthy non-smoking individuals can benefit from acute nicotine use, other studies may investigate whether smokers will receive the same benefit for their vision when they are using nicotine gum to quit smoking. We believe our study can contribute to the development of the field. Having reviewed related work, we now present the main body of our research.

## Methods and Analysis

### Aims

#### Study 1—Contrast Sensitivity Function

The first study will not be conducted simultaneously with the second study because it is a within-subject, and we cannot exclude the possibility of desensitization among individuals; therefore the participants enrolled in this first study will not participate in the second study. The primary aims for the contrast sensitivity function (CSF) measurements are investigating whether any of three conditions (placebo, 2-mg, or 4-mg doses) (a) influence visual performance for low, middle, and high spatial frequencies (ranging from 0.2 to 20 cycles per degree); (b) whether there is a dose dependency on contrast sensitivity; (c) whether BMI (we intend to select a pool of individuals with different BMIs) influence the results; (d) if there are differences related to sex; and (e) whether there is a serum cotinine concentration dependency of contrast sensitivity. Exploratory aims will test effects by age, height, weight, and years of education.

#### Study 2—Cambridge Colour Test

The primary aims of this second study period are investigating whether any of three conditions (placebo, 2-mg, or 4-mg doses) (a) influence chromatic contrast discrimination for the S-, M-, and L-wavelength sensitive cones (blue, green, and red, respectively) using the Trivector subtest; (b) influence the position and direction (e.g., larger or more circular ellipses indicate worse discrimination) of an MacAdam chromatic discrimination ellipse for on an achromatic background (Ellipse 1); (c) determine whether there is a dose effect; (d) determine whether there is an interaction between the dosages and direction of the chromatic contrast discrimination; (e) are the results related to BMIs; (f) are different with regard to sex; and (g) determine whether there is an interaction between cotinine concentration and chromatic contrast discrimination. Exploratory aims will test effects by age, height, weight, and years of education.

### General Study Design

The ENIGMA-Vis is designed to be a randomized, double-blind, and placebo-controlled three-period trial (RBR-46tjy3) to investigate the effects of nicotine gum administration on vision. The diagram is shown in Table 1. This trial comprises three main gum administrations (placebo, 2-mg, and 4-mg doses). Participants will be followed up at 4 weeks, and the assessments will be performed in week 0 (baseline) to week 3. Female participants will be tested outside their luteal phase.

**TABLE 1:** Measures by timepoint for the schedule of enrolment, interventions, and assessments.

	**Study period**					
	**Enrolment**	**Allocation**	**Post-allocation**			**Close-out**
**Timepoint**	**−*t*_1_**	**Week 0**	**Week 1**	**Week 2**	**Week 3**	**40 weeks**
**ENROLMENT**						
Eligibility screening	X					
Informed consent	X					
Neuropsychological measures		X				
Allocation		X				
**INTERVENTIONS**						
Nicotine gum 2 mg			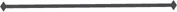			
Nicotine gum 4 mg			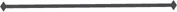			
Placebo gum			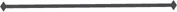			
**ASSESSMENTS**						
(Sex, age, level of education, height, weight, body mass index, serum cotinine)		X				
Contrast sensitivity measures (*n* = 50)			X	X	X	X
Color vision measures (*n* = 50)			X	X	X	X
Secondary outcomes			X	X	X	X

### Participants

**FIGURE 1:**
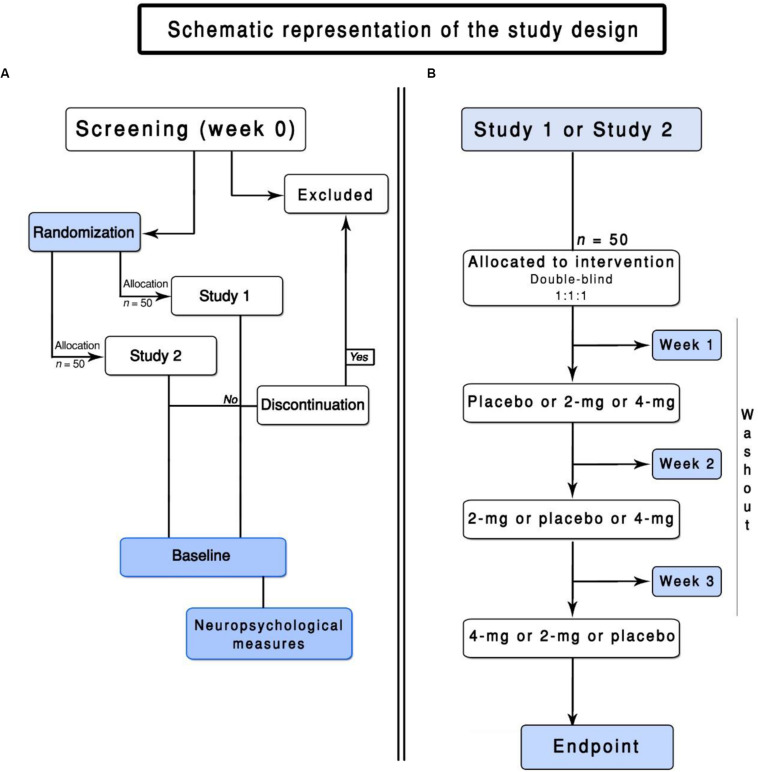
Flowchart of the ENIGMA-Vis. **(A)** represents the screening of the participants and the general design of this trial. **(B)** represents the flowchart of Study 1 or Study 2.

Healthy non-smokers from 18 to 40 years are eligible for the study. We intend to recruit participants over the phone, radio, websites, and newspaper advertisements. An initial screening for eligibility will be done over the phone, 30 days before the start of the study. Potential participants will be invited to schedule a baseline enrollment (Figure 1). After signing an informed consent, the participants will be assessed for eligibility. Inclusion and exclusion criteria are presented below.

### Eligibility Criteria

An investigator, on the basis of the criteria reported below, will determine eligible participants.

#### Inclusion Criteria

All of the participants must sign written informed consent before entry into the study, fulfill the criteria for never smokers (smoked < 15 cigarettes per lifetime; Pomerleau et al., 2004), and be willing and able to comply with the scheduled visits and other trial procedures. The participants should have normal dental status and chewing ability. Finally, the participants would be required to be diagnosed free of ocular diseases by an ophthalmologist during the previous 12 months. All of the participants should have normal or corrected-to-normal vision as determined by visual acuity of at least 20/20 (logMAR ≤ 0.0, using the FrACT; Bach, 1996). All of the participants will be screened for color blindness using the 24-plate edition of the Ishihara test (Ishihara, 1972). More than three errors will be considered a failure (Fernandes et al., 2020). All of the participants will be asked to abstain from caffeine-containing products beginning at midnight the evening prior to the measurements (Fernandes et al., 2018a). The participants will be assessed for caffeine dependence or withdrawal using self-reports. The participants will be assessed for second-hand smoke. The Smoke Scale for Adults (SS-A; Misailidi et al., 2014) will be used as this is a valid, reliable, and practical scale for adults.

#### Exclusion Criteria

Exclusion criteria will be <18 or >40 years old, current history of neurological disorder, cardiovascular disease, pregnant or suspected of being pregnant, a history of head trauma, and a history of contact with such substances as solvents, caffeine addiction or deprivation, and current use of medications that may affect visual processing and/or cognition (e.g., benzodiazepines or antidepressants). Importantly, other acute or chronic conditions will be defined using the *Structured Clinical Interview for Diagnostic and Statistical Manual of Mental Disorders*, 5th edition (American Psychiatric Association, 2013) that may bias the results of this trial will be considered as an exclusion criterion. Individuals with a score of >16 in the SS-A will be excluded. Individuals who participated in other clinical trials in the previous months will be excluded. Finally, neuropsychological testing will be conducted to identify the possibility of cognitive impairment. The tests will include the Mini-Mental State Examination (Folstein et al., 1975) and the Trail Making Tests (Tombaugh, 2004). Individuals with scores ≤ 24 on a paper-based Mini-Mental State Exam (Folstein et al., 1975) and scores below the normative data for age and education in the Trail Making Tests (cutoffs based on the normative data for age and education) will be excluded (Tombaugh, 2004).

### Baseline Measures

All of the baseline measures will be assessed in the allocation phase (see Table 1) at the laboratory. The baseline measures of the participants will include age, sex, height, weight, BMI ($\text{BMI}=\,\frac{\text{Weight}}{{\text{Height}}^{2}}$), and serum cotinine levels. Also, for female participants, information about the menstrual cycle and pregnancy will be assessed. Serum cotinine samples will be collected as a screening to avoid previous nicotine use (biological markers) using 10 ng/ml as a cutoff (Duque et al., 2017). The serum sample will be taken randomly selecting (or selected in order to ensure a balanced representation of gender and BMI) ≥ 50 participants and stored at or below −20°C. They will be measured using a microplate enzyme immunoassay kit, according to the manufacturer’s instruction. Serum cotinine will also be used as secondary outcomes (see “Outcome Measures” section).

### Randomization/Stratification and Blinding/Masking

We will use lists to classify, identify, and separate participants according to randomization, allocation, blinding of outcomes, initials of the participants, and adverse effects.

#### Randomization

Participants will be randomized in accordance with a 1:1:1 permuted block randomization generated by a free resource for researchers^1^. Allocation will be made according to the Cochrane guidelines (Higgins et al., 2019). The main purpose of using a computerized randomization program is to minimize confounding factors and avoid differences between the participants in Study 1 and Study 2 (e.g., similar, or approximately, number of males and females). Fifty participants will be allocated to Study 1, and 50 participants will be allocated to Study 2. All participants will be designated with their initials and a random number by a researcher who is unaware of the condition assignment.

#### Blinding/Masking

The study coordinator will generate the allocation sequence, and two researchers will administer the nicotine gum, but they will be blind with regard to the dosage. Two different researchers will conduct the statistical analyses. They will be unaware of the condition analyzed. The participants will be interviewed at the end of the study (by the selected researchers) to determine if the blinding was broken. If the blind is broken, additional analyses without the unblinded cases will be conducted to test the effects of unblinding. Some participants might be able to tell which dose they received on the basis of physical alterations; however, malaise will be assessed at the end of each period session.

### Safety, Dropouts, and Adherence

The nicotine gum administration can act in the area postrema (chemoreceptor zone). This structure is involved in the emetic mechanisms and can cause nausea (Changeux, 2018). Because nicotine is a vasoactive substance, some participants may report a headache, giddiness, and sweating. Other adverse events can be sore throat due to menthol. Nevertheless, as reported by Naser et al. (2011) and Varghese et al. (2011), the nicotine gum administration is safe, and the adverse events are not related to all participants.

The participants will be asked at the end of each session whether they had adverse events such as “tingling,” “nausea,” and “headache” among others and asked about the intensity (1 = none to 4 = strong) and whether this effect could be related to the nicotine gum. Dropouts will be considered if the individual (1) misses two consecutive visits during the study; (2) does not complete all of the nicotine gum administration; (3) presents any severe adverse events during the trial; and (4) expresses the wish to stop participation for any reason. Retention strategies will be employed to ensure adherence and reduce dropouts, such as accessibility of the investigators, flexibility with the visit scheduling (not exceeding 1 week between measures), transportation to our laboratory, and expense reimbursement.

#### Intervention

**FIGURE 2:**
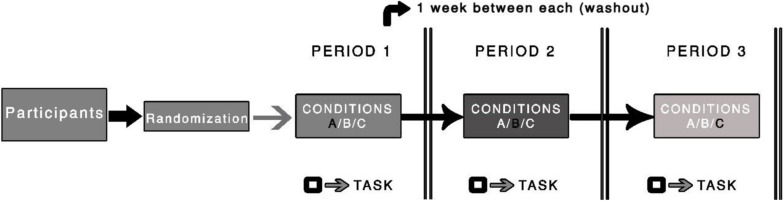
Illustration of the procedure used in the randomized clinical trial. The black square symbol represents the testing procedure (i.e., task). A washout period of one week between periods will be used.

This trial will follow the assumptions proposed by Russell et al. (1976), where the average peak of plasma nicotine is expected to occur between 15 and 45 min after the beginning of the chewing. The tests will be performed in this time window. Two dosages (2-mg and 4-mg) of nicotine gum (Nicorette, GlaxoSmithKline, and Wales) and one placebo gum, with the same taste, texture, and similarity to the nicotine gum (e.g., levomenthol, mint oil, and winter-fresh essence; producing tingling on the gums, the same sensations produced by active nicotine gum) will be used in this study. The choice for using these dosages (2-mg and 4-mg) is based on the commercially available options for the icy mint option. In addition, we opted to pick the two lowest dosages initially to minimize side effects. The participants will be asked to gently chew the gum for 15 min, while maintaining the same speed and intensity before the tests are performed (Figure 2). We will instruct them to minimize the possibility of bias although, to our knowledge, no study has reported an association between speed or intensity of chewing and the amount of nicotine absorbed (for details, see Benowitz et al., 2009). During this time, the participants will be allowed to read recreational material, but they will not be allowed to text or read emails. Members of the research team will monitor the participant to ensure that they chew according to instructions.

After 5 min of chewing, the participants will dark-adapt for 10 min. Next, they will be light-adapt to the corresponding display for 3 min. During these 3 min, they will be familiarized with the corresponding test using suprathreshold stimuli. This procedure will ensure that all the participants are in the same photopic adaptation state and understand the instructions. When the testing begins, 18 min will have passed since they started chewing the gum, and they will dispose of the gum.

### Sample Size

There were no previous reports of sample size estimation from a meta-analysis or equivalent study. Because of the lack of this information, the effect size will be estimated using the minimal meaningful effect size (0.05) (Sullivan and Feinn, 2012) and the power will be used considering a common value in clinical trials (Charan and Biswas, 2013). Considering a two-sided test with an alpha value of 0.01 (or 0.02 for a one-sided test) and a power of 85% (or 0.85), the sample size would require at least 42 individuals to complete the three periods for each study. As this trial is a single group model, with an anticipated 20% attrition rate, our main intention is to enroll 50 individuals for each study (Study 1, *N* = 50; Study 2, *N* = 50). Although our sample size is larger than that of previous studies using these tests (Andrade et al., 2019; Fernandes et al., 2019a,b), it is important to provide an estimation that covers our outcomes (e.g., BMIs and cotinine).

### Outcome Measures

The primary outcomes of this study are about the effects of nicotine on achromatic contrast sensitivity at the various spatial frequencies (Study 1) and on chromatic contrast discrimination using the Trivector threshold values and the various elliptical parameters like angle of the major axis, major axis length, axis ratio, and area (Study 2). For Study 1, the higher the curve, the better the discrimination ability. For Study 2, the area of the ellipse is a general index of chromatic discrimination. That is, the smaller the ellipse, the better the discrimination ability. Provided that there is a statistically significant main effect or interaction, results will be related to the statistical analysis: an improvement of 10% will be considered as meaningful difference for each spatial frequency (Study 1) and for each parameter of the CCT (Study 2). The primary outcome will be assessed after the three sessions are completed. As previously noted, we did not observe learning or practical effects when using Metropsis software (Fernandes et al., 2019a) and the CCT (Fernandes et al., 2020). The secondary outcomes are related to demographic data (effects of age, years of education, height, and weight), BMI, and gender effects. Serum cotinine levels (ng/ml) after each session (placebo, 2 mg, and 4 mg of nicotine gum) will be used as predictors of response (for each visual test) in a multivariate regression analysis. Data will be double-checked and stored at an open access repository (figShare; available upon request).

### Material and Stimuli

The stimuli will be presented on a gamma-corrected 19-inch LG CRT monitor with 1,024 × 786 resolution and a 100-Hz refresh rate. Stimuli will be generated using a VSG 2/5 video card (Cambridge Research Systems), which will be run on a Precision T3500 computer with a W3530 graphics card. All of the procedures will be performed in a room at 26 ± 1°C. The room lights will be extinguished and so the only light sources will be the computer monitors. The experimenter’s monitor will be positioned so that it is outside of the participant’s field of view. Implementation and calibration procedures (gamma-correction) will be performed with software and hardware provided by the Cambridge Research Systems (a ColCAL MKII photometer). The participant’s head will be stabilized using forehead and chin rests. The participants will be instructed to look at the central cross-shaped fixation point (for Study 1) or the center of the CCT background pattern (for Study 2). They will be informed that once the stimulus is presented, they can move their eyes as required to identify which side has the grating or the gap position on the Landolt ring.

#### Study 1—Contrast Sensitivity Function

The Metropsis software (Cambridge Research Systems Ltd., Rochester, United Kingdom) will determine contrast sensitivity. The stimuli for the CSF will be linear, vertically oriented, sine-wave gratings with spatial frequencies ranging from 0.2 to 20.0 cycles per degree (cpd). We will constantly check the outcomes, using the experimenter’s monitor, to observe any floor or ceiling effects of the spatial frequencies. The stimuli consist of equiluminant gratings with dimensions of 5° of a visual angle and will be presented on the monitor at 2.5° spatial offset from the central cross-shaped fixation point (Fernandes et al., 2019b).

Small sample sizes are common in perception research owing to the repeated measures design. However, some researchers still debate whether small sample sizes are reliable and generalizable. The Metropsis software represents a reliable tool to investigate contrast sensitivity and does not require multiple applications and does not suffer variation over time within-subjects. This tool can be used in small sample sizes and still provide reliable data as we previously observed (Fernandes et al., 2019a). Psychometric function of the Metropsis software (e.g., employing a rigorous criterion of almost 80% correct responses for each spatial frequencies) is one of its main advantages. Measures of reproducibility were undertaken (Fernandes et al., 2019a), and values above or below the reported means for each spatial frequency used will be considered as statistically significant differences.

#### Study 2—Cambridge Colour Test

The CCT (Cambridge Research Systems Ltd., Rochester, United Kingdom) will determine chromatic contrast discrimination. The CCT stimulus is a colored Landolt ring displayed within a differently colored background. The position of the opening in the ring is presented randomly in one of four positions; up, down, left, and right. The chromatic contrast of the ring is varied until a threshold is obtained. In our setting, the “ring” will have an opening of 1.25° of visual angle at 3-m viewing distance. To ensure that the break in the ring is identified based only chromatic information, luminance noise is added by subdividing the background and stimulus into small circles randomly varying in size (between 2.8° arcmin and 5.7° arcmin in diameter) and randomly varying in luminance (between 8 and 18 cd/m^2^, in 2 cd/m^2^ of increments). Three different stimuli will be used to measure thresholds along the protan, deutan, and tritan lines of confusion through the background. Thresholds for these three stimuli are determined primarily by the L-, M-, and S-cone, respectively (Regan et al., 1994; Mollon and Regan, 2000). The background is achromatic and located at *u*′ = 0.1977, *v*′ = 0.4689 (CIE 1976 chromaticity diagram). This background will also be used for the Ellipse 1 trial, measuring it with eight vectors. The Ellipse subtest measures the chromatic discrimination ellipse for three different backgrounds, located along the same tritan confusion line; however, in this study, only the achromatic background will be evaluated because of time constraints. The total time to complete both tests is approximately 12 min (6 min each; for more details, see Almustanyir et al., 2020). The CCT represents a cognitively simple test, is easily grasped by individuals, and provides reliable results (Paramei and Oakley, 2014).

### Procedures

It is worth reiterating that different participants will take part in each study. Fifty participants will be randomly allocated to Study 1, and another 50 participants will be allocated to Study 2 (see Figure 1).

#### Study 1—Contrast Sensitivity Function

Before the start of the session, instructions will be provided. The participants will perform a short training with high contrast stimuli at each spatial frequency to familiarize with the procedure and to avoid misunderstanding (part of the light-adapting, see section “Intervention”). Accuracy over speed will be emphasized. The Metropsis software incorporates a check on the validity of the data by using catch trials (suprathreshold stimuli) to minimize random responding.

Measurements will be performed binocularly at a distance of 150 cm from the computer monitor. The participants respond whether the grating is presented on either the left or right side of the computer screen. They will be instructed to respond even if they are not sure. A number of catch trials (commonly used in perception studies to investigate whether or not the participant grasped the task) will be randomly intermixed with the test trials to detect bias. The catch trials will be presented after every 30 trials for each of the tested spatial frequency at a contrast 10% above the current contrast for that spatial frequency; however, the catch trials will not be used to compute the threshold. Apart from the catch trials, no auditory or visual feedback will be used, as providing feedback is uncorrelated to the observer’s response (Herzog and Fahle, 1997; Bach and Schäfer, 2016).

**FIGURE 3:**
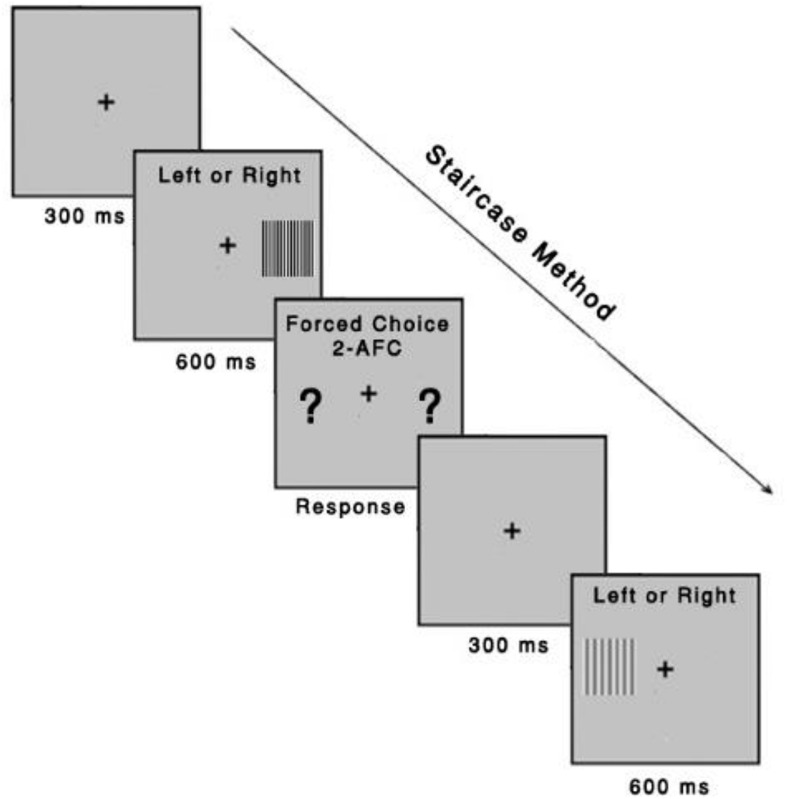
Contrast sensitivity function task. The task was to identify, using a remote control response box, whether the gratings were presented on the left or right side of the computer screen. Each stimulus had an exposure time of 600 ms, with an intertrial interval of 300 ms. The Metropsis algorithm randomizes spatial frequencies (low, medium, and high) and contrast values.

A three-down one-up logarithmic staircase with dynamic steps is used to derive a contrast threshold with a level of accuracy of target detection of 79.4% on a psychometric function (Fernandes et al., 2019a). Initially, the contrast values appear at the suprathreshold level, for which we expect correct responses. After three consecutive correct responses, contrast decreases until an incorrect response. After every incorrect response, contrast increases. The Metropsis software computes the threshold after 12 contrast reversals have occurred at each spatial frequency. The order of the spatial frequencies is randomized within a session. Each stimulus has an exposure time of 600 ms. After responses were observed, the next trial starts after 300 ms. A higher CSF value indicates better discrimination (Figure 3). The CSF test can be performed in approximately 15–20 min.

#### Study 2—Cambridge Colour Test

**FIGURE 4:**
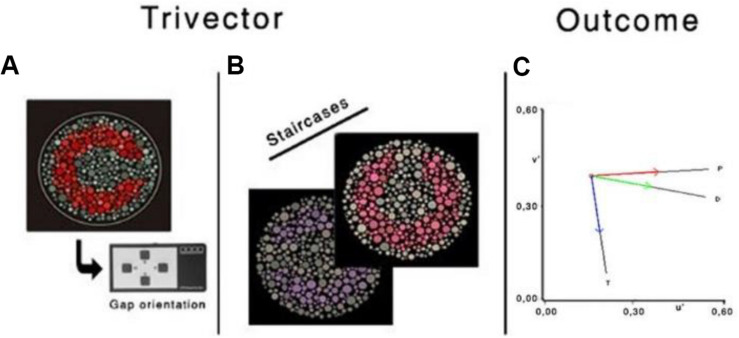
Illustration of the chromatic targets **(A,B)**, Landolt “ring,” embedded in the luminance noise background. **(C)** Protan (P), deutan (D), and tritan (T) vectors in the CIE 1976 *u*′*v*′ chromaticity diagram. Reprinted from Fernandes et al. (2020) with permission from © The Optical Society.

The subject will be instructed to identify the orientation of the Landolt ring gap—presented randomized in one of the four positions [four-alternative forced choice (4-AFC)]—by pressing the corresponding button of the response box (CT6, CRS). If the location of the gap is not seen, then they should guess. The chromatic contrast of the Landolt ring varies relative to that of the background, using an adaptive staircase procedure (Figure 4). The specific rules are the chromatic contrast is halved (24%) after a correct response and doubled (48%) following an incorrect response or no response (within the allocated response time), until the first reversal, and 8% for the remaining reversals (in either direction). Periodically, a catch-trial target at maximum saturation is presented. It constitutes approximately 10% of the stimuli. Apart from the catch trials, no auditory or visual feedback will be used, as providing feedback is uncorrelated to the observer’s response (Herzog and Fahle, 1997; Bach and Schäfer, 2016). The test stops after six staircase reversals for each vector; chromatic contrast discrimination threshold (in *u*′*v*′ units) is computed as the average of the chromaticities corresponding to the six reversals. For each of the three confusion lines, the CCT algorithm implements two interleaved staircases presented in a random order using a weighted a one up/one down staircase rule, with a ratio of 1/3 to converge on the 75% threshold. Accuracy over speed will be emphasized in the instruction. The response box will be held by the participant with both hands, and the thumbs will be used for the button pressing. The time allowed for observers to respond will be 6 s. Both Trivector and Ellipse subtests should be completed by a participant in approximately 12 min (inside the peak concentration of nicotine).

### Pupil Effects

Because it was unclear as to whether the reduction in pupil size (Erdem et al., 2015) was due to nicotine or some other chemical in cigarette smoke, we carried out a pilot study (Columbus) on the effects of nicotine gum on pupil size. The main results indicated nicotine produced a small reduction in pupil size [baseline, 3.28 mm (± 0.24); 2-mg, 3.20 mm (± 0.17); and 4-mg, 3.15 mm (± 0.20)]. The change in pupil size is small. The maximum reduction in retinal illumination would be equivalent to a filter that transmits 92% of the light, which is relatively equal to the transmittance of a clear spectacle lens after accounting for light loss due to reflections. Based on these findings, we believe this reduction in retinal illumination is unlikely to affect the results.

### Statistical Analysis

For each study, the distribution of data will be assessed, including measures of central tendency and measures of dispersion. Distributions for each group will be compared using the Monte Carlo method for skewness and kurtosis, and the cutoff value will be ± 1.96, suggesting that departure from normality will not be observed (Antonius, 2003; Tabachnick and Fidell, 2007). We will both compare outcomes from skewness and kurtosis with the Shapiro–Wilk values. Data will be first plot or graph and then analyzed accordingly to the study. Statistical analysis will be performed using SPSS 25.0 and MATLAB R2018b^2^.

#### Study 1—Contrast Sensitivity Function

For the contrast sensitivity analysis, we will use a multivariate analysis of variance (MANOVA), with eight dependent variables (thresholds of each spatial frequency; continuous variables) and one independent, within-subject variable (condition ∼ three levels). *Post hoc* testing will be conducted using discriminant analysis.

#### Study 2—Cambridge Colour Test

For the Trivector subtest of the CCT, we will use a one-way MANOVA, with three dependent variables (thresholds for protan, deutan, and tritan; continuous variables), and one independent, within-subject variable (condition ∼ three levels). For the Ellipse subtest, we will conduct a separate MANOVA for Ellipse 1 with two dependent variables (angle and area; continuous variables) and one independent, within-subject variable (condition ∼ three dosage). A moderation or mediation analysis (using 5,000 bootstrapping resamples) will be conducted to determine whether the area, length, angle, or ratio is a better predictor of any results of the CCT.

#### Additional Analyses

Missing data will be considered as random (MAR), and the maximum likelihood estimation (MLE) will be used. Under the MAR assumption, MLE appears to represent an interesting and unbiased approach, even to continuous and discrete variables (Dziura et al., 2013). Nevertheless, we will analyze individuals with missing data and without missing data. We expect to have no more than 5% of missing data and differences between with and without missing data.

Exploratory analysis to investigate whether sex, age, and level of education are predictors of response will be performed. Multivariate regression analysis will be used to investigate whether demographics would be predictors of the effects of nicotine gum. We will also conduct a separate regression analysis to investigate whether or not cotinine serum levels would be predictors of visual performance. These variables can be also used as mediators of the interaction between conditions.

Finally, adverse events will be presented as percentages in each condition. Cochran’s *Q* test will be conducted to investigate the effectiveness of the masking and blinding (on this analysis, we will calculate the ratio between errors and correct responses regarding the condition in which the participant believed was applied).

## Discussion

ENIGMA-Vis aims to investigate short-term effects of nicotine gum on vision in a population of healthy non-smokers. The findings obtained here will allow us to undertake the controlled investigation of nicotine effects on vision. We believe that this study design, altogether with the use of reliable tools such Metropsis software (CSF) and the CCT, will additionally allow us to observe the acute (short-term) effects of the nicotine. As follows, we can address an important but understudied area: the role of nicotine on vision. With this trial, we intend to open new avenues on visual research. Caution should be taken with the generalization of our findings, however.

### Anticipated Results

We expect, based on our hypothesis, that nicotine could lead to an improvement in visual processing, with the higher dose producing the largest improvement.

As observed in our past studies (de Almeida et al., 2018; Fernandes et al., 2018b,c; Silva et al., 2020), tobacco addiction resulted in early-stage visual processing impairments. However, smoking comes in the form of (i) smoke absorption and (ii) the consumption of numerous health-damaging compounds (nicotine is one of the numerous existing components) (Silva et al., 2020). In the mammalian brain, nAChR isoforms are expressed on mesolimbic and nigrostriatal neurons. Because about one-fourth of the brain is involved in visual processing and neurotransmitters such dopamine, GABA, and glutamate are also involved, it is not hard to explain that transient nicotine administration can have effects on vision (Balfour and Munafò, 2015). Note that the expression of nAChRs in the brain does not imply agreement of intakes = effects. This may be explained because the intake of nicotine gum is relative to the maximum amount and other factors such peak concentration, age, BMI, and gene expression (Benowitz, 2010; Balfour and Munafò, 2015). In light of these factors, our attempt is to control confounding factors and investigate the extent to which nicotine can improve vision.

### Strengths and Limitations of This Trial

We have proposed a different approach, and this research is a first step toward a more profound understanding of the effects of nicotine on vision. To our knowledge, this is the first trial attempting to investigate it. We must mention that this study still cannot be placed as a final step of the investigations of visual processing, and because of this, we cannot simply state that our findings should be labeled as the last step of investigations using nicotine gum on vision. We need, first, to investigate visual processing in terms of contrast sensitivity and chromatic contrast discrimination using stimuli, which could involve all the three visual pathways. Visual processing may be a door to cognition. Understanding the improvements may help promote policies that seek to act directly for individuals with impairments in visual processing before these impairments progress to higher-order cognition (Butler et al., 2005; Tacca, 2011).

The strengths of this study include having a rigorous controlled clinical trial with a larger sample size. Also, the use of reliable tools for investigating visual processing can be argued as a strength of this research. Nevertheless, some concerns regarding our study design should be underscored. First, although the sample size is larger than used in previous studies, we do not have a solid base to conclude that it is a sufficiently large sample size. Second, due to the high cost, we will not be able to provide an extensive pharmacological and physiological explanation of the results. This is particularly the case for the measuring the cotinine levels. It may be too costly to obtain cotinine levels for all participants (*n* = 100), and so these levels will be based on randomly selecting (see section “Baseline Measures”). Third, because fixation is not controlled and eye movements are not monitored, it is unknown as to whether any differences in search patterns after ingesting nicotine would be related to any differences in the sensitivity. However, studies showing that practice has minimum effects on these two tests suggest that changes in search patterns would not affect sensitivity (Costa et al., 2006; Fernandes et al., 2019a, 2020). Any search patterns would be relatively simple, shifting one’s gaze horizontally for the CSF and both horizontally and vertically for the CCT. Previous findings on eye movements indicate that saccadic latencies are shorter after ingesting nicotine, and so any improvement in saccadic eye movements would likely be reflected in their response times rather than in their sensitivity (Bowling and Donnelly, 2010). Although the total time to complete the CSF and the CCT is different (e.g., 15–20 and 12 min, respectively), both tests will be completed within the time for the average peak concentration of nicotine (< 45 min).

Given these limitations, our trial will be an initial, but important, investigation about the effects of nicotine on vision.

In summary, we believe that our results will provide motivation for follow-up studies. Our results may also help understand why certain groups (e.g., people with schizophrenia) demonstrate both visual dysfunctions and elevated rates of tobacco addiction.

## Ethics Statement

The studies involving human participants were reviewed and approved by Ethics in Research of the Health Sciences Center of Federal University of Paraiba (Trial register number: RBR-46tjy3). Written informed consent was not provided because subjects have not yet been recruited. Written informed consent will be obtained from all of the participants indicating that they had been informed of all aspects of the trial at the beginning of the trial.

## Author Contributions

TF and NS conceived and drafted the study protocol. JH helped draft, revise, and proofread the manuscript. NA and MA helped with technical and theoretical work. TB, SR, GS, JJS, and JBS were involved in the design and research methodologies. MO, GG, EL, MG, MJ, and MM helped in providing outcome measures and planning data collection and analyses. All authors listed have made a substantial, direct and intellectual contribution to the work, and approved it for publication. All authors agreed to be accountable for all aspects of the work, ensuring accuracy and integrity of any part of the work.

## Conflict of Interest

The authors declare that the research was conducted in the absence of any commercial or financial relationships that could be construed as a potential conflict of interest.

## Footnotes

1 www.randomizer.org

2 www.mathworks.com
